# Rare presentation of emphysematous pyelonephritis due to faulty catheter insertion

**DOI:** 10.1002/ccr3.6251

**Published:** 2022-08-18

**Authors:** Sarah Honjo, Taiju Miyagami, Mayu Suzuki, Arisa Ito, Toshio Naito

**Affiliations:** ^1^ Department of General Medicine Juntendo University Faculty of Medicine Tokyo Japan

**Keywords:** emphysematous pyelonephritis, *Klebsiella pneumoniae*, necrotizing infection

## Abstract

Emphysematous pyelonephritis is a severe necrotizing infection that occurs predominately among individuals with diabetes mellitus. The mortality rate is >10% only medical therapy. We report a case of emphysematous pyelonephritis caused by *Klebsiella pneumoniae*, precipitated by faulty catheter insertion. The patient recovered after the replacement of catheter and administration of antibiotics.

## 1 INTRODUCTION

**FIGURE 1:**
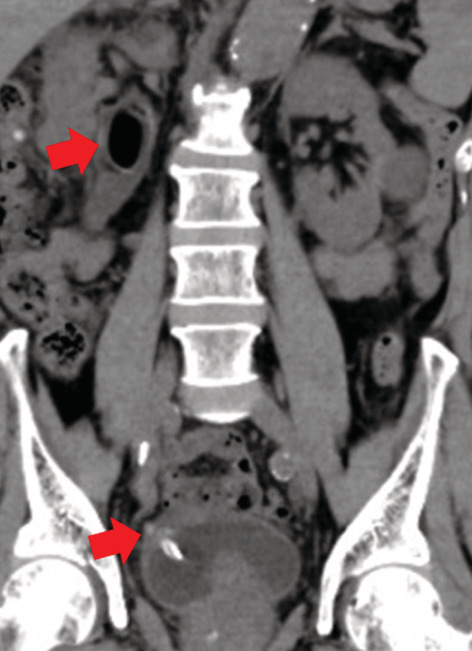
Non‐contrast‐enhanced computed tomography of the abdomen (coronal section) showing a bladder catheter embedded in the bladder wall (arrow) and showing air in the ureter (arrow)

An 83‐year‐old man with diabetes mellitus (HbA1c, 6.6%) and an indwelling urinary catheter, which had been inserted for benign prostatic hyperplasia, presented to the emergency department with a fever of 38°C, chills, and abdominal pain. These symptoms appeared after the replacement of the urinary catheter at his primary care clinic. On admission, all vital signs, other than body temperature, were stable. Physical examination revealed mild left costovertebral angle tenderness. Non‐contrast computed tomography of the abdomen showed an embedded catheter in the bladder wall and air in the ureter (Figure 1) suggesting emphysematous pyelonephritis (EPN) as described in a former report where emphysema in the ureter was classified as EPN.^1^

The bladder catheter was replaced, and piperacillin/tazobactam was administered empirically then switched to ceftriaxone after *Klebsiella pneumoniae* was detected in blood and urine cultures. The patient was treated for 14 days and made a full recovery.

A study on patients with EPN, of which more than 80% had pre‐existing diabetes mellitus, revealed that approximately 10% of patients who had only administered medical therapy died.^2^ EPN should be considered in cases of fever and abdominal pain after bladder catheter insertion.

## AUTHOR CONTRIBUTIONS

SH, TM, MS, AI, and TN have made substantial contributions to conception and design, or acquisition of data, or analysis and interpretation of data. SH, TM, MS, AI, and TN are involved in drafting the manuscript or revising it critically for important intellectual content; have participated sufficiently in the work to take public responsibility for appropriate portions of the content; and agreed to be accountable for all aspects of the work in ensuring that questions related to the accuracy or integrity of any part of the work are appropriately investigated and resolved.

## CONFLICT OF INTEREST

All authors have no pertinent conflict of interest to report for this manuscript.

## ETHICAL APPROVAL

None.

## CONSENT

Written informed consent was obtained from the patient to publish this report in accordance with the journal's patient consent policy.

## Data Availability

None.
